# Particle therapy using protons or carbon ions for cancer patients with cardiac implantable electronic devices (CIED): a retrospective multi-institutional study

**DOI:** 10.1007/s11604-021-01218-1

**Published:** 2021-11-15

**Authors:** Takayuki Hashimoto, Yusuke Demizu, Haruko Numajiri, Tomonori Isobe, Shigekazu Fukuda, Masaru Wakatsuki, Haruo Yamashita, Shigeyuki Murayama, Shigeyuki Takamatsu, Hiroyuki Katoh, Kazutoshi Murata, Ryosuke Kohno, Takeshi Arimura, Taeko Matsuura, Yoichi M. Ito, Japan Radiological Society multi-institutional study group

**Affiliations:** 1 Department of Radiation Medical Science and Engineering, Faculty of Medicine, Hokkaido University, North 15 West 7, Kita-ku, Sapporo, Hokkaido 060-8638 Japan; 2 Department of Radiology, Hyogo Ion Beam Medical Center, 1-2-1 Kouto, Shingu-cho, Tatsuno, Hyogo Japan; 3 Department of Radiation Oncology, University of Tsukuba, 1-1-1 Tennodai, Tsukuba, Ibaraki Japan; 4 QST Hospital, National Institutes for Quantum and Radiological Science and Technology, 4-9-1 Anagawa, Inage-ku, Chiba, Japan; 5 Shizuoka Cancer Center Hospital, 1007 Shimonagakubo, Nagaizumi-cho, Sunto-gun, Shizuoka, Japan; 6 Department of Radiation Therapy, Kanazawa University Hospital, 13-1 Takara-machi, Kanazawa, Ishikawa Japan; 7 Department of Radiation Oncology, Gunma University Graduate School of Medicine, 3-39-22 Showa-machi, Maebashi, Gunma Japan; 8 Department of Radiation Oncology, Kanagawa Cancer Center, 2-3-2 Nakao, Asahi-ku, Yokohama, Kanagawa Japan; 9 National Cancer Center Hospital East, 6-5-1 Kashiwanoha, Kashiwa, Chiba Japan; 10 Department of Accelerator and Medical Physics, National Institute for Quantum and Radiological Science and Technology, 4-9-1 Anagawa, Inage-ku, Chiba, Japan; 11 Medipolis Proton Therapy and Research Center, 4423, Higashikata, Ibusuki, Kagoshima Japan; 12 Faculty of Engineering, Hokkaido University, Kita 13, Nishi 8, Kita-ku, Sapporo, Hokkaido Japan; 13 Biostatistics Division, Clinical Research and Medical Innovation Center, Hokkaido University Hospital, Kita14, Nishi5, Kita-Ku, Sapporo, Hokkaido Japan

**Keywords:** Proton beam therapy, Carbon ion therapy, Cardiac implantable electronic devices, Electrical reset, Secondary neutron

## Abstract

**Purpose:**

To evaluate the outcomes of particle therapy in cancer patients with cardiac implantable electronic devices (CIEDs).

**Materials and methods:**

From April 2001 to March 2013, 19,585 patients were treated with proton beam therapy (PBT) or carbon ion therapy (CIT) at 8 institutions. Of these, 69 patients (0.4%, PBT 46, CIT 22, and PBT + CIT 1) with CIEDs (64 pacemakers, 4 implantable cardioverter defibrillators, and 1 with a cardiac resynchronization therapy defibrillator) were retrospectively reviewed. All the patients with CIEDs in this study were treated with the passive scattering type of particle beam therapy.

**Results:**

Six (13%) of the 47 PBT patients, and none of the 23 CIT patients experienced CIED malfunctions (*p* = 0.105). Electrical resets (7) and over-sensing (3) occurred transiently in 6 patients. The distance between the edge of the irradiation field and the CIED was not associated with the incidence of malfunctions in 20 patients with lung cancer. A larger field size had a higher event rate but the test to evaluate trends as not statistically significant (*p* = 0.196).

**Conclusion:**

Differences in the frequency of occurrence of device malfunctions for patients treated with PBT and patients treated with CIT did not reach statistical significance. The present study can be regarded as a benchmark study about the incidence of malfunctioning of CIED in passive scattering particle beam therapy and can be used as a reference for active scanning particle beam therapy.

**Supplementary Information:**

The online version contains supplementary material available at 10.1007/s11604-021-01218-1.

## Introduction

Pacemakers (PMs) are surgically implanted medical devices that generate electrical impulses to treat irregular or stalled heartbeats, and more than 1 million people worldwide have PMs implanted annually [1]. The incidence of malignancies is on a rising trend, and approximately two-thirds of all patients with malignancies receive radiation therapy (RT) at some point during the treatment of their disease in the United States and European countries. Although this percentage is approximately half of that in Japan, the ratio of cancer patients who have been treated using RT has increased in step with the rapidly growing proportion of elderly people [2]. Treatment modalities for malignant diseases, such as surgical resection and chemotherapy are often unsuitable for patients with cardiac implantable electronic devices (CIEDs), such as PMs, implantable cardioverter defibrillators (ICDs), and cardiac resynchronization therapy defibrillators (CRT-Ds), because of reduced cardiac function. As a result, increasing numbers of patients with CIEDs will require RT in the treatment of their malignant diseases [3].

Particle therapy using protons or carbon ions is a radiation modality which has excellent dose distributions to the target and reduces or eliminates unnecessary radiation to normal tissue. There are 107 particle beam institutions in the world including 24 in Japan and 40 in the U.S.A. in February 2021 [4].

To date, many investigators have reported on the clinical effects of RT on CIED functioning [3, 5, 6]. The secondary neutron radiation is a concern during high-energy (≥ 10 MV) photon radiotherapy and particle therapy even when the CIED is situated outside of the treatment field [7–9]. Hazards linked to the effects of secondary neutrons on CIEDs may cause clinical problems in CIED-wearing patients. Current data about particle therapy with CIEDs carriers originate from in vitro experiments and clinical studies that are from single institutions and usually include a limited number of patients [9–12]. Recently, recommendations from AAPM have stated that particle beam therapy should be avoided for patients with CIED since it produces secondary neutrons [13]. The Japanese Society for Radiation Oncology (JASTRO) and The Japanese Circulation Society (JCS) also published official guidelines for radiotherapy in patients with cardiac implantable electronic devices [14]. In the JASTRO/JCS guideline, patients who would receive particle beam therapy are classified in the high-risk patient category for patients who receive X-ray energy ≥ 10 MV, electron beam energy ≥ 20 MeV, the estimated dose to the main body of CIED ≥ 10 Gy, or who are PM-dependent and with a history of ventricular fibrillation or ICD intervention. In the guidelines, it is stated that “proton beams also generate secondary neutrons, and CIED malfunctioning during proton beam therapy (PBT) has been reported; therefore, proton beams are also high risk. Fewer secondary neutrons are generated by carbon-ion beams than with protons, but carbon ions are still considered to be high risk.” Careful preparation before radiotherapy and strict checking of the function of the CIEDs are required for high-risk patients in clinical practice based on the guidelines.

Regarding the dose outside the treatment field, Xu et al. have published a comprehensive review of the physical aspects [15]. They summarized, that in photon treatment it was clear by the early 1990s that (1) the photon dose outside the treatment field decreases exponentially with increasing distance from the field edge, (2) the neutron dose is relatively independent of the distance from the field edge, (3) the dependence of the photon dose outside the treatment field on both depth and beam energy is very weak, (4) the dependence of the neutron dose on depth and beam energy is very strong and (5) the dose outside the treatment field increases with increasing field size. They also reviewed reports of passive scattering particle beam therapy and summarized that (1) the neutron dose decreased with the distance from the field edge and (2) the neutron yield in the patient increases with field size whereas the neutron yield from the treatment head, scattering devices, modulators, and patient-specific apertures, or compensators depends on the ratio of the field size and aperture opening. It is as yet uncertain, however, whether the distance between the beam and the CIED and the irradiation field size are associated with malfunctions of CIED in actual patients who received passive scattering particle beam therapy.

There is hope that new types of particle beam therapy, scanning-type therapy, would reduce the risk of malfunction of the CIEDs since the scanning-type can reduce the incidence of secondary neutrons compared to conventional passive-type particle beam therapy [15–17]. In the present study, we report the results of a retrospective, multi-institutional survey of the incidences of malfunction of CIED in conventional passive-type particle beam therapy for cancer patients with contemporary CIEDs. The relationships between the malfunction and type of particle beam, the field size, and the distance from the field edge were investigated where the relevant data was available. We hope this report will provide a benchmark for passive-type particle beam therapy for patients with contemporary CIEDs to be compared with scanning-type particle beam therapy in the future.

## Materials and methods

### Facilities and treatment modalities

To conduct a multi-institutional, retrospectively ascertained cohort of cancer patients treated with PBT or carbon ion therapy (CIT), we asked all PBT and CIT centers in Japan about the possibility for them to participate in the study in 2013. At that time, there were 8 PBT centers and 3 CIT centers (One hospital had both PBT and CIT rooms). Each room was counted as one center for each modality. Six PBT centers and 3 CIT centers agreed to participate in the study. Treatment using scanning technology had already started at some facilities. However, all patients with CIEDs in this study were treated with the passive scattering type of particle beam therapy. The energies of the therapeutic beams were from 115 to 235 MeV for PBT, and 140–400 MeV/n for CIT. The relative biological effectiveness (RBE) was set at 1.1 for PBT and 3.0 for CIT in all facilities.

The research protocol for this study was first approved by the institutional review board (IRB) of Hokkaido University Hospital (IRB number 014-0046) and then by the IRB of each participating institution, which granted a waiver of informed consent from study participants due to the retrospective nature of the study. Particle therapy records were reviewed for treatment-specific variables including irradiation site, prescription dose and fractionation, modality, and energy.

### Patients

From April 2001 to March 2013, 19,585 patients were treated in eight Japanese institutions (Supplemental Table 1). There were 10,550 patients treated with PBT and 9,035 treated with CIT. Of these, 46 in PBT, 22 in CIT, and one patient who received both PBT and CIT were patients with CIEDs. There were 54 males and 15 females. The age of the 69 patients was from 60 to 97 with a median age of 81 years. Cancer types, pathology, and clinical stage of these patients are detailed in Table 1.

**Table 1:** Particulars for patients and cancers

Characteristic	Patients
Modality	
Proton	46
Carbon	22
Proton+Carbon^a^	1
Gender	
Male	54
Female	15
Cancer type	
Liver cancer	26
Hepatocellular carcinoma	21
(Stage I, II, IIB, IIIA, IVA)	(11, 5, 3, 1, 1)
Cholangiocarcinoma	1
(Stage I)	(1)
Unclassifiable	4
Lung cancer	20
Non-small cell lung cancer	18
(Stage IA, IB, IIA, IIIA)	(8, 5, 1, 3)
(Relapsed lymph-node)	(1)
Metastatic, primary unknown	2
Prostate cancer	15
Adenocarcinoma	15
(Stage I, II, III)	(6, 4, 5)
Bone and soft tissue sarcoma	4
Iliac chondrosarcoma	1
(Stage III)	(1)
Sacral osteosarcoma	1
(Stage IVA)	(1)
Thigh pleomorphic cell sarcoma	1
(Stage IIIB)	(1)
Gluteus leiomyosarcoma, metastatic, primary unknown	1
Head and neck tumor	3
Choroidal malignant melanoma	1
(Stage IIB)	(1)
Nasal malignant melanoma	1
(Stage IVB)	(1)
Submandibular gland carcinoma^b^	1
(Stage IV)	(1)
Pancreas cancer	1
Adenocarcinoma	1
(Stage III)	(1)

^a,^^b^The primary submandibular gland carcinoma was treated by proton beam therapy and a metastatic tumor relapsed at the orbit was treated by carbon ion therapy in this patient

**Fig. 1:**
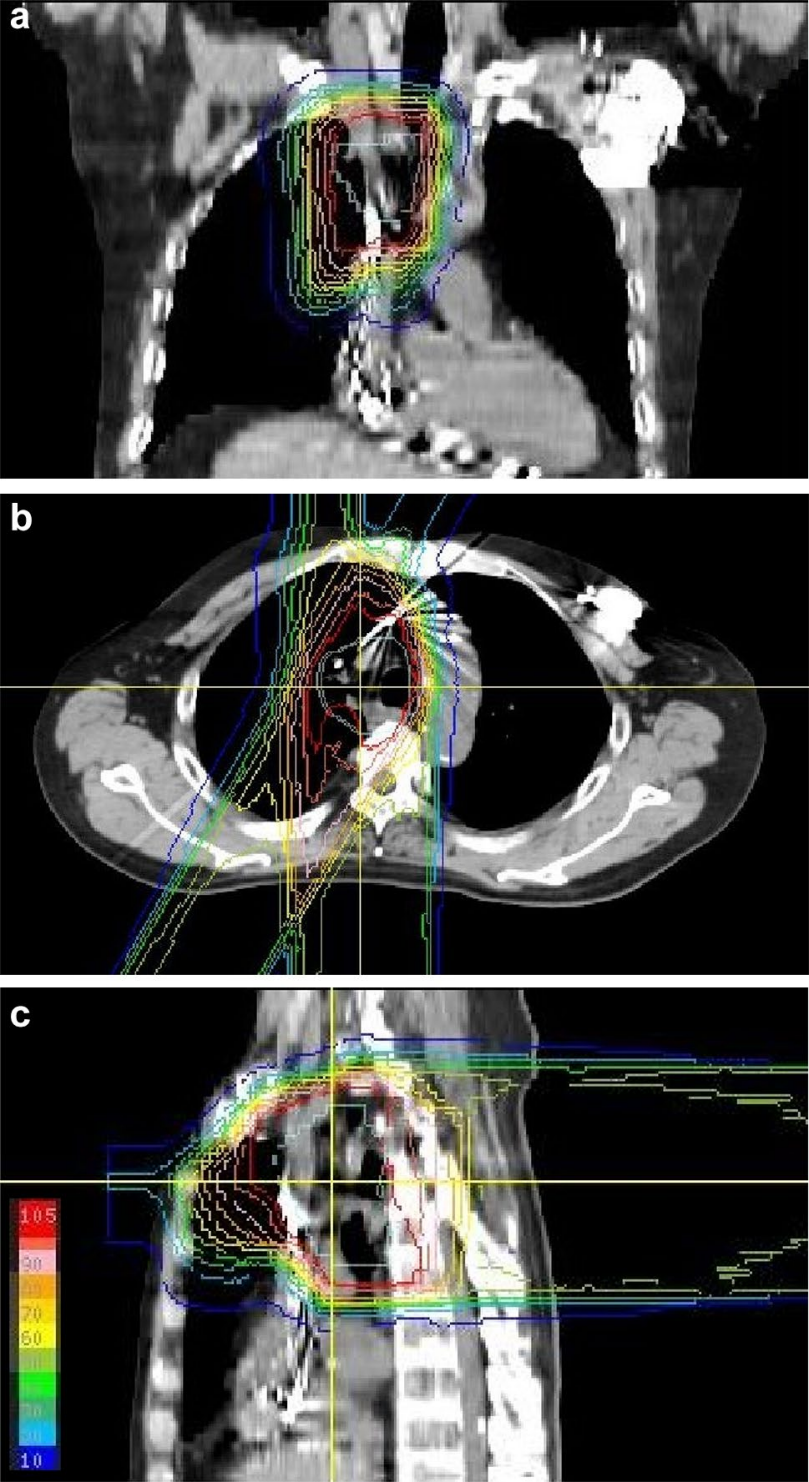
A representative image set for the lung cancer patient with a pacemaker which was inserted at the left infraclavicular region: coronal view (**a**), axial view (**b**), and sagittal view (**c**). No device malformation occurred in this patient

Patients had implanted PMs in 64, ICDs in 4, and a CRT-D in 1 patient. A representative imaging data set of a patient with implantable PM near the treatment field is shown in Fig. 1. Venders of CIED and insertion site of the CIED are listed in Table 2.

**Table 2:** Cardiac implantable electronic device particulars, manufacturer, and site of insertion

Characteristics	Number
Device type	
Pacemaker	64 (93%)
ICD	4 (6%)
CRT-D	1 (%)
Vender	
Medtronic	21
St. Jude medical	10
Biotronic	5
Guidant	5
Vitatron	5
Boston scientific	1
Guidant	1
Pacesetter	1
Sorin	1
Unknown	19
CIED insertion site	
Left infraclavicular region	57
Right infraclavicular region	6
Unknown	6
Total	69

*ICD* implantable cardioverter defibrillator, *CRT-D* cardiac resynchronization therapy defibrillator

It is well known that the out-of-field dose decreases exponentially with the distance from the field edge, and we investigated the distance of the CIED from the field edge in the 20 patients with lung cancer. The maximum field size in each patient was also investigated to determine whether there would be any relationship between the malfunction and the field size. There were 47 patients in PBT and 23 patients in CIT in whom the maximum irradiation field size was available.

### Statistical analysis

Categorical data were analyzed by Fisher’s exact test or chi-square test. Proportion differences and 95% confidence intervals between groups were estimated by the adjusted Wald method [18]. The relation between the incidence of the malfunction and the maximum irradiation field size was assessed using a Cochran–Armitage trend test. All statistical analysis was performed by JMP pro 14 (SAS Institute Inc., Cary, NC). Data were considered statistically significant at values of *p* < 0.05.

## Results

### Clinical events

The prescribed dose ranged from 36.3 to 88.0 Gy (RBE) with the median dose at 70.0 Gy (RBE) in PBT treatments and 28.0–77.0 Gy (RBE) with the median dose at 57.6 Gy (RBE) in CIT treatments. Fraction sizes varying between 2.0 Gy (RBE) and 6.6 Gy (RBE) in PBT treatments, and between 2.2 Gy (RBE) and 22.5 Gy (RBE) in CIT treatments.

When a patient was treated twice at different times, we counted this as two treatments. There were 47 treatments in PBT and 25 treatments in CIT. Modality and treatment sites are listed in Table 3. In all the 72 treatments, the full irradiation dose was delivered as scheduled without any major cardiac event.

**Table 3:** Incidence of device malfunctions by treatment site and treatment modality

Treatment site	Treatments and malfunctions^a^			
	Proton		Carbon	
	Treatments	Malfunctions	Treatments	Malfunctions
Upper body	17	3	8	0
Head and neck	1	1	3	0
Lung	16	2	5	0
Middle body	20	3	8	0
Liver	20	3	7	0
Pancreas	0	0	1	0
Lower body	10	0	9	0
Prostate	9	0	6	0
Bone and soft tissue	1	0	3	0
Total	47	6	25	0

^a^When one patient was treated twice at different times, this was counted as two treatments

### Device malfunctions

There were 6 device malfunction events in the 72 treatments. All of the 6 (13%) were among the 47 PBT treatments, and no device malfunctions were observed in the 25 CIT treatments. Proportion differences and 95% confidence intervals between the PBT and CIT malfunctioning were 0.128 (− 0.022 ~ 0.227), which was not statistically significant (*p* = 0.105).

The treatment sites of all the treatments with PBT and CIT and those with the 6 malfunction events are shown in Table 3. There was no significant difference in the distribution of treatment site, upper body, middle body, and lower body, or between PBT and CIT. The incidence was not different for the upper body group (3/17, 18%) and for the middle/lower body group (3/30, 10%) in the PBT treatments.

Details of the malfunctions observed in this study are shown in Table 4. Four (6%) of the 64 patients with PM, 1 (25%) of the 4 with ICD, and 1 (100%) of the 1 patient with CRT-D experienced device malfunction during the particle beam therapy. Three patients experienced multiple device malfunctions; 2 experienced 2 resets of PM and 1 experienced 3 over-sensings of the ICD. Patient No.6 experienced malfunction during PBT for a primary salivary gland tumor but did not experience a malfunction during CIT for an orbital metastatic tumor.

**Table 4:** Details of device malfunctions observed in the patients

No	Diagnosis	Stage	Age/ gender	Device type/insertion site	Dose/fraction [Gy (RBE)/fr]	Modality/ energy	CIED model	Malfunction	Outcome
1	HCC	cT1N0M0 St. I	79/M	PM/ Unknown	66.0/10	Proton/ 155 MeV	St. Jude/ Affinity DR 5330	Reset at 39.6 Gy (RBE)	Recurrent-free at 31 mo Died suddenly of heart disease
2	NSCLC	cT2bN0M0 St. IIA	75/F	PM/ Left IR	72.6/22	Proton/ 200 MeV	Unknown	2 Resets at 24.0, 66.0 Gy (RBE)	Died of cancer at 17 mo
3	HCC	rT1N0M0 St. I	68/M	CRT-D/ Left IR	72.6/22	Proton/ 155 MeV	Medtronic/ Insync III Marquis 7279	Reset	Alive with disease at 4 mo
4	NSCLC	cT2aN0M0 St. IB	74/M	ICD/ Left IR	70.0/35	Proton/ 155 MeV	Biotronik/ Lexos DR	3 Over sensings	Recurrence-free at 110 mo Died of other disease
5	HCC	cT3bN0M0 St. IIIB	76/M	PM/ Left IR	67.5/25	Proton/ 210 MeV	St. Jude/ Integrity μ SR	2 Resets at 18.9, 27.0 Gy (RBE)	Died of cancer at 17 mo
6^a^	SMC	cT4bN0M0 St. IVB	69/F	PM/ Left IR	70.2/26	Proton/ 150 MeV	Biotronik/ Philos DR	Reset at 27.0 Gy (RBE)	Alive with disease at 21 mo

*CIED* cardiac implantable electronic device, *HCC* hepatocellular carcinoma, *NSCLC* non-small cell lung cancer, *SMC* submandibular carcinoma, *PM* pacemaker, *IR* infraclavicular region, *CRT-D* cardiac resynchronization therapy defibrillator, *ICD* implantable cardioverter defibrillator

^a^Patient No. 6 received carbon ion therapy for the orbital metastatic tumor later but not experienced malfunction with the carbon ion therapy

The relationship between the distance between the edge of the irradiation field and the CIED in the 20 patients with lung cancer is shown in Supplemental Table 2. No malfunction was observed in 12 treatments in which the distances from the edge of the treatment fields were within 15 cm whereas 2 malfunctions were observed in 5 treatments with the distances 15–20 cm from the CIED. No malfunction was observed in 3 treatments in which the distances were 20–25 cm.

**Fig. 2:**
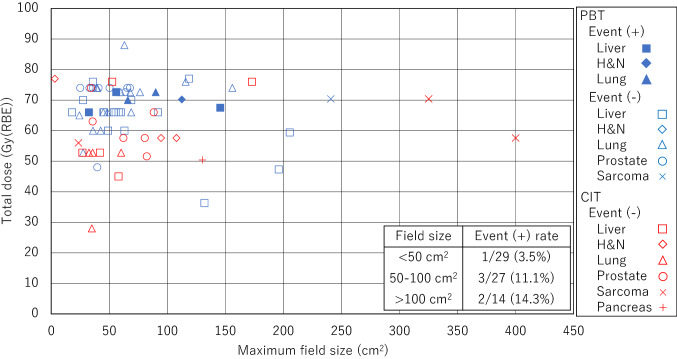
Relationship between the maximum field size, prescribed dose, and CIED malfunctions

The relationship between the maximum field size, the prescribed dose, and the CIED malfunction is shown in Fig. 2. The malfunctions were observed in 0/24 (0%), 6/46 (13%) for the total dose of 60 Gy (RBE) or less and more than 60 Gy (RBE), respectively. Total doses of more than 60 Gy (RBE) had a higher event rate but the test to evaluate trends was not statistically significant (*p* = 0.064). The malfunctions were observed in 1/29 (3%), 3/27 (11%), and 2/15 (13%) for the field size of 0–50 cm^2^, 50–100 cm^2^, and > 100 cm^2^, respectively. The larger field sizes had higher event rates but the test for trends was not statistically significant (*p* = 0.196) (Fig. 2).

No permanent device malfunctions were observed. One 79-year-old patient (No.1 in Table 4) died suddenly of heart disease at 31 months following the PBT. There had been no malfunction episode after the PBT and we determined that the malfunction of the CIED during the PBT treatment was not related to the death.

## Discussion

Published CIED studies with particle therapies using PBT or CIT have been limited primarily to single-center reports of a small number of patients. To our knowledge, this is the first multi-institutional, and the largest retrospective series reported for the effect of particle therapy on CIEDs functioning.

In the present study, we report the outcomes of a retrospective, multi-institutional survey of particle therapy using PBT and CIT for cancer patients with contemporary CIED. Device malfunctions occurred in 13% of PBT treatments and no permanent device malfunctions were observed. Neutron-producing forms of the radiotherapy with a beam energy ≥ 10 MV photon or particle therapy are more likely to be associated with device malfunctions. Several reports have been published on the effects of photons with high energies > 10 MV [3, 5, 7, 19]. Grant et al. have shown that malfunctions occurred at a rate of 21% in 71 treatments of neutron-producing photon therapy but with no event occurring among 178 treatments of non-neutron-producing photon therapy [7]. Gomez et al. have reported that 5 (11%) patients experienced device malfunctions among 42 patients with CIED receiving PBT [10]. Our results and the Gomez et al. results suggest that the incidence of device malfunctioning is not more frequent in PBT than in neutron-producing photon therapy. This is consistent with the results of the Yonai et al. study which found that the neutron ambient dose equivalent in passive particle radiotherapy is equal to or smaller than that in the photon radiotherapy [20].

A significant effect of radiotherapy on device functioning leading to electrical resets even though outside the irradiation field with minimal measured radiation exposure has been reported [7, 21]. Only resets and over-sensing were observed in our study. The most frequent pattern of device malfunction was electrical resets. Gomez et al. have reported that in 6 cases malfunctions occurred in 5 patients receiving PBT, of these 5 were resets and one was due to an elective replacement indicator (ERI) [10]. Since the timing of the ERI signal was consistent with that predicted before treatment, the ERI was thought not to have been influenced by the PBT. Ueyama et al. reported that resets occurred in 2 out of 7 patients with PM receiving PBT [12]. These reports suggest that resets are the most common type of malfunction in patients with CIED receiving PBT.

The frequency of malfunction is higher in ICD and CRT-D comparing to PM in this study. There have been a few reports about these devices other than PM and its sensitivity to radiation [22, 23]. Kapa et al. have pointed out that the recommendations by a vender suggested that ICDs may be 5–10 times more sensitive to radiation damage than PM since operation instructions are stored in random access memory that may be more readily damaged by radiation. Grant et al. have shown that ICD was associated with a higher risk of malfunction in PBT (*p* = 0.02) compared with PM by a univariate analysis in photon therapy although it was not significant in a multivariate analysis (*p* = 0.052) [7]. Gomez et al. reported that 2 (7%) of 28 patients with PM and 3 (21%) of 14 patients with ICD experienced device malfunctions in PBT [10]. In the present study, malfunction was evident in 4 of the 65 (6%) PM, 1 of 4 (25%) ICD, and 1 of 1 (100%) CRT-D after particle therapy. This suggests that the ICD may be associated with higher risks of malfunction than the PM.

Grant et al. have suggested that treatment of the abdomen/pelvis is associated with higher risks of malfunction than treatment of the chest/head/neck/total body among the neutron-producing photon group [7]. Zaremba et al. have also shown that tumors below the diaphragm are associated with device malfunctions in photon therapy [3]. Different from this, Gomez et al. have reported that all resets occurred in patients who received thoracic PBT [10]. In our series, there was no difference in the incidence between the upper body treatments and middle/lower body treatments. There is a need for further investigation of the relationship between the site of irradiation and the incidence of device malfunction.

Xu et al. have summarized that unlike photon therapy, the dose outside the main irradiation field in passive scattering particle beam therapy is entirely due to neutrons generated in nuclear interactions and the neutron sources originate either from the treatment head or in the patient [15]. They suggested that for neutrons generated in the treatment head, the materials and specific arrangements of the beam shaping devices are predominant and that the neutron dose is dependent on the facility and on the setting for the different patient fields. A considerable portion of the beam may be stopped in the patient-specific aperture, which causes the neutron dose to be dependent on the ratio of the field size and aperture.

Measurements and Monte Carlo simulations have suggested that the neutron dose decreased with the distance from the edge of the irradiation field in passive scattering particle beam therapy [15]. A comprehensive experimental study using an anthropomorphic phantom by Mesoloras et al. verified that the neutron dose decreases with increasing aperture size [24]. The distance between CIED and the edge of the irradiation field was not apparent in 20 patients with lung cancer in our series. Since many patients must have been treated with patient-specific apertures, the influence of the distance from the field edge may have been obscured in the present study.

Zacharatou-Jarlskog et al. showed that the neutron yield from the treatment head decreased with increasing field size but the neutron yield in the patient increases with treatment volume or the field size [25]. Therefore, the relationship between neutron generation and field size must be a complex one depending on the balance of contribution from the treatment head and from the patient. We have observed that the larger field sizes had a higher event rate but the test to evaluate the trend was not statistically significant. This result does not contradict the physical complexity of the neutron dose production in passive scattering particle beam therapy, however.

The difference between PBT and CIT is not conclusive statistically and further cases need to be added. There are several reasons which may make CIT safer than PBT. One reason is the lower total physical doses in CIT than in PBT. We have found that total doses lower than 60 Gy (RBE) were associated with a lower incidence of malfunctions in this study. If we do not use weighting factors for the physical absorbed dose, i.e., RBE = 3.0 for CIT and RBE = 1.1 for PBT, to equalize the biological difference between PBT and CIT, the total physical absorbed dose is much lower than in CIT than in PBT. This can be a reason why fewer secondary neutrons are produced in CIT than in PBT. Also, the previous dosimetric studies suggested that CIT produces fewer secondary neutrons than PBT even for the same physical absorbed dose in Gy [20, 26].

The present study has several limitations. First, the number of patients at risk is small. Another limitation is the retrospective design. It is known that minor errors may be discovered after the radiotherapy by a detailed analysis of the timestamps corresponding to event occurrences recorded by the CIED. Not all the devices were investigated thoroughly after the particle therapy in this study, and this may have resulted in underestimates of the incidence of device malfunction. Data of the distance between the particle beam equipment and the CIED were not always available. Especially for patients with diseases in the pelvis, CT scans was not performed at the level of CIEDs so an evaluation of the distance was not possible. We have not measured neutron dose distributions and electromagnetic waves in the treatment room which should also have been investigated as a possible source of the malfunctions of CIEDs.

There was no institution which used scanning beams in this study. It has been suggested that scanning particle beam therapy can reduce the secondary neutron volume dramatically [16, 17, 20, 26]. Seidansaal et al. have reported that there were no device malfunctions among 32 patients (10 PBT and 22 CIT) with CIED in their investigation [27]. Yonai et al. have suggested that the neutron dose will be reduced with an order of magnitude with active scanning PBT and CIT [17, 20]. The present study can be regarded as a benchmark study about the incidence of malfunction of CIED in passive scattering particle beam therapy and a reference for active scanning particle beam therapy.

Use of neutron-producing radiation is the principal risk factor in device malfunctions. Particle beam therapy generates secondary neutrons and the risk of malfunction of CIED cannot be ruled out. These errors may occur even when the device is far from the irradiation field. However, particle therapy is less invasive and provides patients with a possible alternative treatment option in some cases. To keep open as many options for cancer treatment as possible, the evaluation of contraindications of particle therapy using protons or carbon ions for cancer patients with CIEDs should be made carefully. And if the particle beam therapy needs to be used, it is necessary to follow the guidelines carefully to reduce the risk of malfunctions of CIED [13, 14, 28].

We have not investigated whether a part of the target volume has been insufficiently covered or a part of the organs at risk has been irradiated with higher doses in patients with CIED because of the limited beam angles near the CIED. Since particle beam therapy requires smaller numbers of treatment portals than photon therapy in general, dose coverage of the target volume caused by the limited irradiation angles near the CIED may possibly be lowered more than those in the photon beam therapy in patients who have cancers near the CIED. More precise work with dose volume statistics is required to investigate these issues.

## Conclusions

Device malfunctions induced by passive scanning particle therapy on CIED are rare, but are an unavoidable and unpredictable phenomenon. Differences in the frequency of occurrence of device malfunctions in patients treated with PBT and in patients treated with CIT did not reach statistical significance. The present study can be regarded as a benchmark study about the incidence of malfunctions of CIED in passive scattering particle beam therapy and a reference for active scanning particle beam therapy.

## Supplementary Information

Below is the link to the electronic supplementary material.

Supplementary file1 (DOCX 25 kb)
