# Supplementary material for: Particle therapy using protons or carbon ions for cancer patients with cardiac implantable electronic devices (CIED): a retrospective multi-institutional study
Source: Jpn J Radiol. 2021 Nov 15;40(5):525–33. doi: 10.1007/s11604-021-01218-1 (PMC9068656; doi:10.1007/s11604-021-01218-1)
Supplement: Supplementary file 1 — Supplementary file1 (DOCX 25 kb) [file 11604_2021_1218_MOESM1_ESM.docx]

**Supplemental Table 1** Number and treatment particulars of patients by institution

| Institution |  | Number of patients | | | |
| --- | --- | --- | --- | --- | --- |
|  | Modality | No. | Subtotal | with CIED | Events* |
| A | Proton | 1430 | 10550 | 9 | 0 |
| B | Proton | 568 |  | 1 | 0 |
| C | Proton | 263 |  | 4 | 0 |
| D | Proton | 2795 |  | 17 | 4 |
| E | Proton | 4327 |  | 14 | 2 |
| F | Proton | 1167 |  | 2 | 0 |
| G | Carbon | 6423 | 9035 | 15 | 0 |
| H | Carbon | 1991 |  | 6 | 0 |
| I | Carbon | 621 |  | 2 | 0 |
| Total |  | 19585 | 19585 | 70 | 6 |
| *Patients who experienced device malfunction during the radiotherapy | | | | | |
| *CIED* cardiac implantable electronic device | | | | | |

**Supplemental Table 2** The distance between the edge of the irradiation field and the CIED in patients with lung cancer

| Distance to field edge | Patient number at risk | | | Number of malfunctions |
| --- | --- | --- | --- | --- |
|  | Total | Proton | Carbon |  |
| 0-5cm | 0 | 0 | 0 | 0 |
| 5-10cm | 5 | 3 | 2 | 0 |
| 10-15cm | 7 | 4 | 3 | 0 |
| 15-20cm | 5 | 5 | 0 | 2 |
| 20-25cm | 3 | 3 | 0 | 0 |
| Total | 20 | 15 | 5 | 2 |
